# Preservation of parathyroid glands during thyroid and neck surgery

**DOI:** 10.3389/fendo.2023.1173950

**Published:** 2023-05-31

**Authors:** Smitha S. Rao, Himagirish Rao, Zia Moinuddin, Anthony P. Rozario, Titus Augustine

**Affiliations:** ^1^ Department of Endocrine and Breast Surgery, Oncology, K.S. Hegde Medical Academy, Nitte University, Mangalore, India; ^2^ Department of Endocrine and General Surgery, St. John's National Academy of Health Sciences, Rajiv Gandhi University of Health Sciences, Bangalore, India; ^3^ Department of Transplant and Endocrine Surgery, Manchester Royal Infirmary, Manchester University Foundation Trust, Manchester, United Kingdom; ^4^ Faculty of Biology, Medicine and Health, Division of Diabetes, Endocrinology and Gastroenterology, Manchester Academic Health Science Centre, University of Manchester, Manchester, United Kingdom

**Keywords:** Parathyroid identification, total thyroidectomy, hypocalcaemia, autotransplant, neck dissection

## Abstract

The parathyroid glands are situated in close proximity to the thyroid gland. They have an important endocrine function maintaining calcium and phosphate homeostasis in the body by the secretion of parathormone (PTH), which is responsible for this function. The parathyroid glands are commonly damaged during thyroid surgeries. This could lead to transient or permanent hypoparathyroidism in 30% of cases. Preservation of the parathyroid glands, is an important and integral part of thyroidectomy and other surgical interventions in the neck. The main principle underlying this is a thorough understanding of parathyroid anatomy in relation to the thyroid gland and other important structures in the area. There can also be significant variation in the anatomical location of the glands. Various techniques and methods have been described for parathyroid preservation. They include intraoperative identification utilizing indocyanine green (ICG) fluorescence, carbon nanoparticles, loupes, and microscopes. The techniques of surgery (meticulous capsular dissection), expertise, central compartment neck dissection, preoperative vitamin D deficiency, extent and type of thyroidectomy are the risk factors associated with damaged thyroids, inadvertent parathyroidectomy and subsequent hypoparathyroidism. Parathyroid Autotransplantation is a treatment option for inadvertent parathyroidectomy. Ultimately, the best way to assure normal parathyroid function is to preserve them *in situ* intraoperatively undamaged.

## Introduction

Surgery of the neck encompasses a wide variety of procedures of varying magnitude. Thyroidectomy is one of the most common procedures for benign disease, whereas neck dissection, is an integral component of malignant disease in the head and neck area. Morbidity associated with neck surgery include bleeding, lymphatic injury, nerve injury, and hypocalcaemia due to hypoparathyroidism. Post-surgical hypocalcaemia can be transient or permanent. Symptoms though transient can be significantly debilitating. Numbness, cramps, tetany, and confusion may require intravenous or multiple doses of calcium. Loss of vascularity can cause transient hypocalcaemia while inadvertent parathyroidectomy will cause permanent hypocalcaemia. Transient hypocalcaemia accounts for up to 18-30% and permanent is restricted to less than 3% of all thyroidectomies. Both transient symptomatic and permanent hypocalcaemia require treatment with permanent hypocalcaemia requiring lifelong oral calcium and active vitamin D3 supplements. Long-term complications include basal ganglia calcifications, renal calcification, carpopedal spasm, cardiac issues, and psychiatric problems (1). 75% of patients with permanent hypoparathyroidism experience significant symptomatology despite treatment and may require admissions. Autotransplant of the parathyroid gland is a reasonable treatment option to prevent permanent hypocalcaemia. There is however unpredictable viability and functionality of the transplanted parathyroid tissue in not an insignificant number of patients (2, 3). Lahey et al. (4) described the first parathyroid autotransplant and Wells published the first series of autotransplants (5). Parathyroid tissue for transplantation can be fresh or cryopreserved. Intraoperative preservations of parathyroid however remains the gold standard to ensure absence of postoperative hypoparathyroidism. Assessment of vascularity in the parathyroid by color assessment is subjective, hence the use of adjuncts to identify the gland (6, 7). Various methods have been found to identify and preserve these glands during surgery. The aim of this paper is to discuss the problem of postsurgical hypocalcaemia and synthesize the available surgical techniques, adjuncts and technological advances in preserving parathyroid function during neck surgery.

## Post-operative hypocalcaemia: the magnitude of the problem

Hypocalcemia features in every discussion on the complications of thyroidectomy. While clinical features can range from the subtle (excessive fatigue, etc.) to the dramatic (stridor, seizures), it is clinically occult in the majority of patients with only biochemical evidence of hypocalcemia and sub-normal S. PTH levels.

Post-operative hypocalcaemia can be immediate, delayed, transient or permanent (**Table 1**).

**Table 1:** Nomenclature of post-op hypocalcemia.

Immediate hypocalcemia	Presentation within 24hrs of surgery
Delayed hypocalcemia	Presentation after 24 hours after surgery
Transient hypocalcemia	Return of normocalcemia within 6 months of surgery
Permanent hypoparathyroidism	Persistent hypocalcemia and hypoparathyroidism for longer than 6 months post-op

It is generally transient and patients mostly recover within a few days after surgery. Various researchers have reported the incidence of transient post-thyroidectomy hypocalcemia between 2% & 51% (8–10). Transient hypocalcemia is seen so frequently after total thyroidectomy that it is now considered a sequel to the procedure and is no longer considered a complication (1). Permanent hypocalcemia as mentioned earlier is rare (0-3.6%) and can be managed with long term calcium and vitamin D replacement (11, 12). Renal failure, basal ganglia calcifications, neuropsychiatric derangements and infections are some of the complications of permanent hypocalcaemia which can have significant morbidity having a negative impact on daily living and quality of life in a subset of patients.

## Risk factors for post-operative hypocalcaemia

It is natural to ascribe post-operative complications to surgeon factors, but as a matter of fact, factors that put the patient at risk can be classified into surgical, anatomical, pathological and even metabolic factors that are independent of surgical anatomy or technique (**Table 2**).

**Table 2:** Factors that influence post-operative hypocalcaemia.

Surgical	Rough tissue handling Intra-operative angiospasm Bleeding in the surgical field Capsular dissection Energy devices Magnification (loupes, etc.) Endoscopic surgery Two or more glands visualised and preserved Inadvertent parathyroidectomy of two or more glands Recurrent goitre Lymph node dissection for cancer	Hinders identification Protective Protective Protective Protective Inferior glands especially at risk
Anatomical	Anomalous location End arteries	
Metabolic	Graves’ disease and thyrotoxicosis Macrodilution General anaesthesia Hypomagnesemia Hypothermia	Hungry bone syndrome

**Figure 1:**
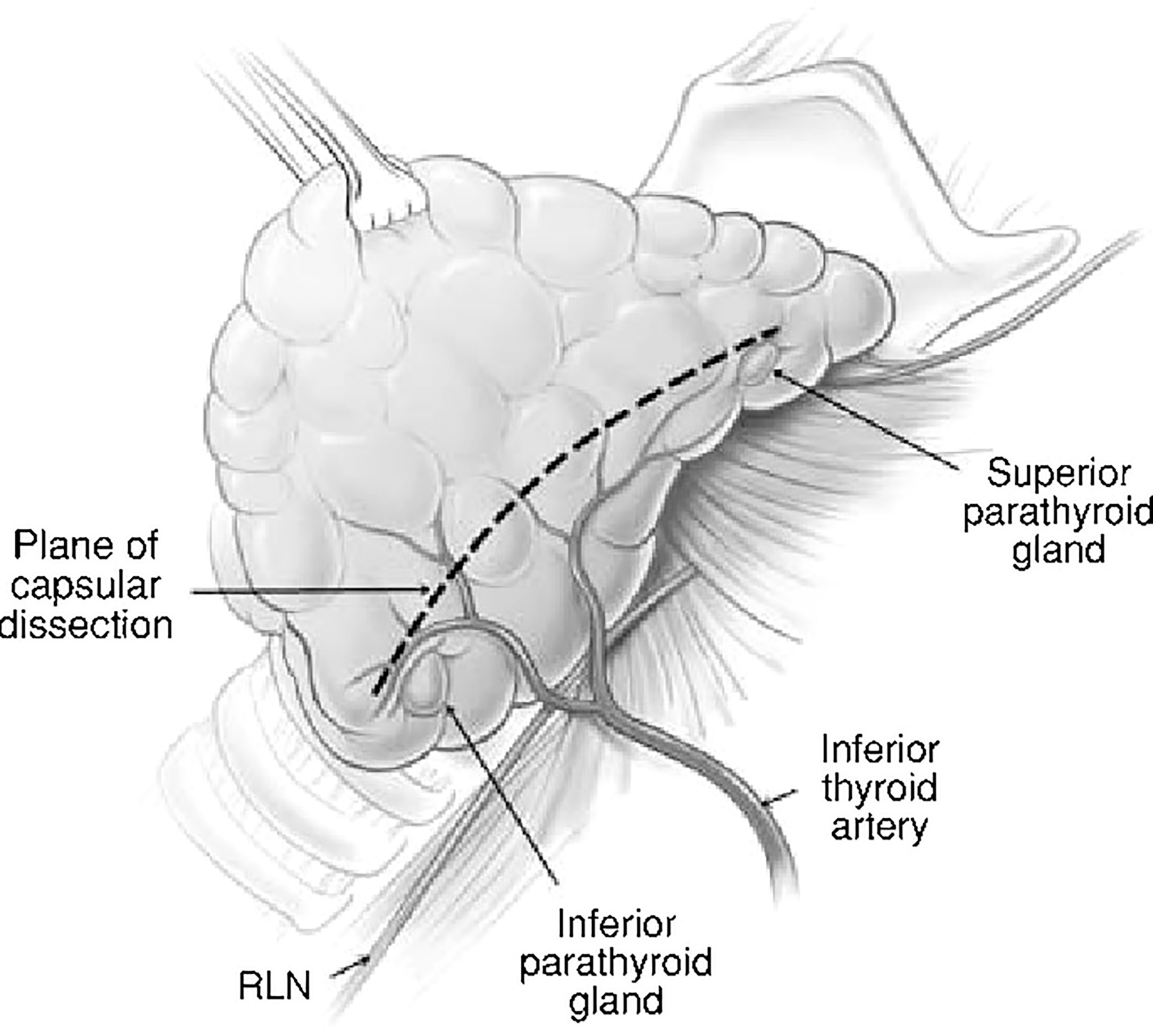
Capsular dissection: the line on the anterolateral surface of thyroid capsule along which dissection should proceed in order to preserve the parathyroids along with their blood supply.

Surgeon factors include tissue handling and dissection (capsular dissection, **Figure 1**), bleeding in the surgical field, use of magnifying aids like loupes and identification of the parathyroid glands on table (13). With the advent of newer surgical approaches with magnification and endoscopic assistance, we are beginning to see the effect of these aids on parathyroid preservation. There have been reports of significant reduction in post-op hypocalcemia with minimal access thyroidectomy (14).

The arterial twigs supplying the glands are end arteries with no collateral anastomoses. Even with the anatomy intact, these vessels might go into spasm post-operatively. Research has revealed that patients with normal S. iPTH levels (intact PTH in circulation) at the end of surgery can develop subnormal levels after surgery (15).

Various researchers have successfully correlated hypocalcemia with the number of parathyroids identified and preserved at surgery. Vidyasagar, et al. (15) estimated the intra-operative and post-operative iPTH levels and correlated these with the number of parathyroid glands which were devascularized or removed during total thyroidectomy. All those patients who had two or more viable and functional glands had normal levels of iPTH, with no significant difference between the intra-operative iPTH levels among them. In those patients with three devascularized glands, the iPTH levels were subnormal, while iPTH was undetectable after devascularization of four parathyroids. In a similar study, Rafferty, et al. (16) correlated post-op hypocalcemia with number of glands found in the specimen after thyroidectomy. The finding of 3 or more parathyroid glands in the specimen correlated with an incidence of 100% for post-operative hypocalcemia, while the incidence rate was 18% or less when two or fewer parathyroid glands were found on the specimen. Histopathological examination of the operated specimen confirms inadvertent parathyroidectomy. When evaluated so, the incidence of unintentional parathyroidectomy ranges from 9% to 19% in different series (17, 18).

Recurrent goitre and revision thyroidectomy is also associated with an increased risk of transient and permanent postoperative hypoparathyroidism (18, 19).

Risk of post-operative hypoparathyroidism and hypocalcaemia is increased in thyroid carcinoma and the procedure of lymph node dissection that may be necessitated (19, 20). Moley and de Benedetti (21) reported that adequate central node dissection is usually associated with compromise of the anatomy and/or blood supply of the parathyroid glands, especially the inferior parathyroids.

In addition to anatomical and surgical reasons, there are other metabolic factors which influence the development of post-operative hypoparathyroidism and hypocalcaemia. Thyroid hormones stimulate osteoclasts and calcium resorption. Post-operative hungry bone syndrome is well-recognised in thyrotoxicosis (17, 22, 23). Hypocalcemia has been shown to occur after other surgeries such as herniorrhaphy as well (22). Macrodilution of the intravascular fluid compartment, general anaesthesia, hypomagnesemia and hypothermia are some of the metabolic factors that might influence post-operative calcium levels (24, 25). With regards to gender, it has been found that this problem seems to affect the females more than males. It is thought that this is because the hormonal response of parathyroids is weaker in females than males (26, 27).

## Parathyroid anatomy: number and location

In an individual, there are usually 4 parathyroid glands which are situated adjacent to the thyroid gland. There is however significant developmental variation in the position of these glands. Historically, Richard Owen identified parathyroid in rhinoceros and Ivar Sandstrom named it ‘glandulae parathyroidae’ (28). The superior parathyroid glands are more anatomically consistent while the inferior parathyroid glands are more variable in position. Embryologically, the inferior parathyroid migrates to a greater extent hence, can be located anywhere between the hyoid bone and the superior mediastinum. This is the reason for the damage of parathyroid most often during surgical procedures in the central compartment of the neck (28, 29). Arterial supply to both superior and inferior parathyroid glands is predominantly from the inferior thyroid artery although there supply sometimes can arise from the superior thyroid artery and the thyroidea ima artery.

## Classification of inferior parathyroid glands

A variety of classifications for the inferior parathyroid glands have been described in the literature.

**Figure 2:**
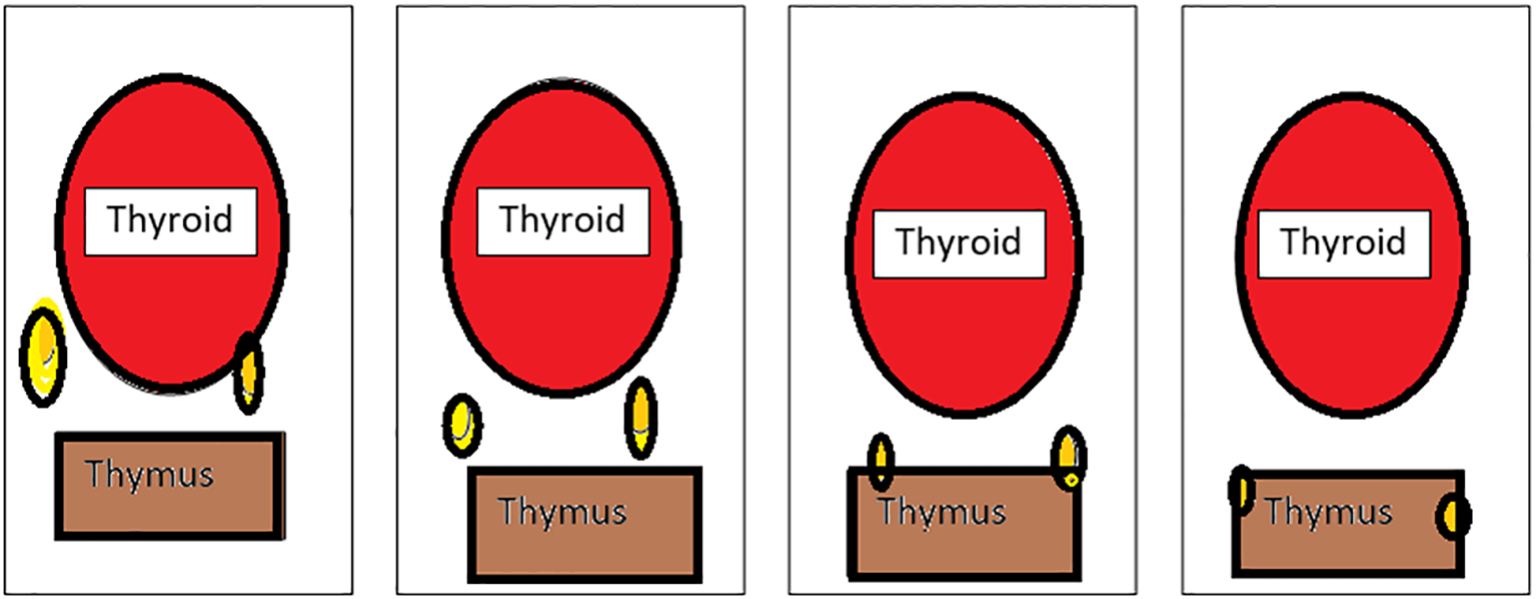
Types of inferior parathyroid (Grisoli)-(1): Usual, (2):Thyrothymic, (3): Superior thymic, (4): Intrathymic.

Grisoli (**Figure 2**) classified the inferior parathyroid according to its relationship with the thymus (28–30).

Group 1 - parathyroid supplied by inferior thyroid artery behind and below the lower lobe of the thyroid

Group 2 - inferior parathyroid in the thyrothymic position placed midway between inferior thyroid lobe and cornua of the thymus

Group 3 - superior thymic parathyroid placed and the cornua of the thymus

Group 4 - intrathymic parathyroid

**Figure 3:**
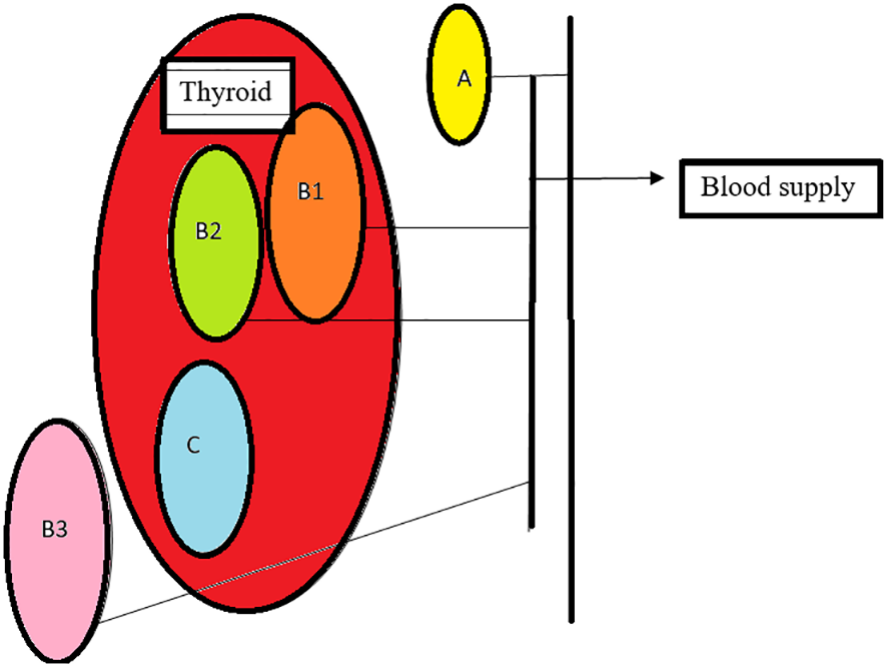
Types of parathyroid: **(A)**- non attachment to thyroid with adequate supply, **(B1)**- light attachment to thyroid with retained supply after thyroid removal, **(B2)**-light attachment to thyroid, changes color, needs auto graft, **(B3)**- blood supply from the thyroid, **(C)**- intracapsular, requires auto transplant.

Based on the relationship between the blood supply of the thyroid and parathyroid, they are classified as follows: Type A- parathyroid supply independent of the thyroid with retained color after thyroidectomy, B1- parathyroid which retains partial blood supply from thyroid and sustains after removal of the thyroid, B2- parathyroid which retains partial blood supply from thyroid and is de-vascularized on the removal of the thyroid, B3- blood supply majorly from the thyroid, difficult to preserve *in situ* C- blood supply completely from the thyroid (30) **Figure 3**

**Figure 4:**
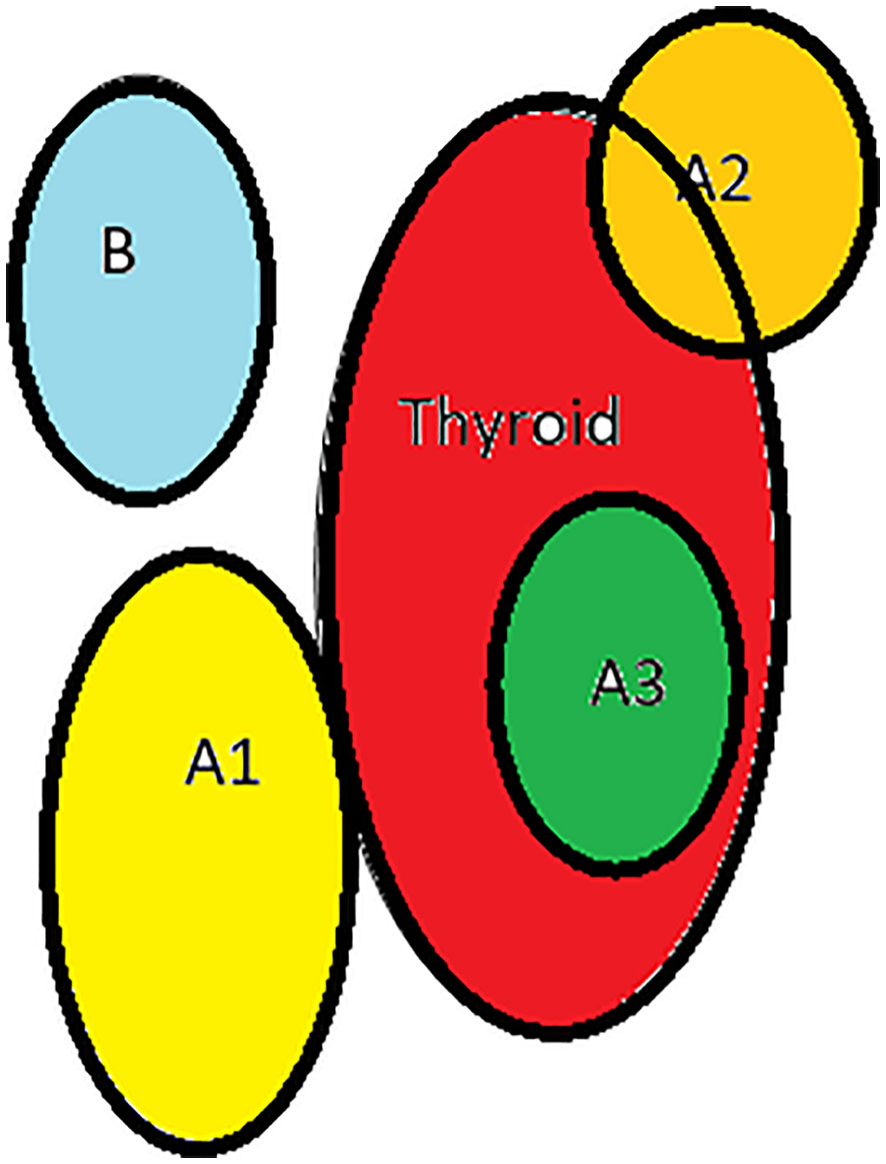
Types of inferior parathyroid (Zhu): **(A1)**-planar attachment, **(A2)**-embedded, **(A3)**- intrathyroid, **(B)**-non close contact.

3. Based on the relationship between thyroid and inferior parathyroid by Zhu, 2 types- A- close contact, B- nonclose contact, A1-planar attachment, A2-embedded attachment, A3- intrathyroidal, B1-around thyroid, B2- intrathymic, B3- supply from thymus and mediastinum **Figure 4**

While these classifications provide anatomic and surgical clues to the variations in parathyroid locations, there are also certain particular locations where they commonly reside.

**-** Around a 1cm circular area around the lower cornu of the thyroid cartilage, 85% of superior parathyroids lie here.
**-** In relation to the recurrent laryngeal nerve (% sign)- superior parathyroids are always posterior to the thyroid while inferior parathyroids anterior to the recurrent laryngeal nerve
**-** Related to the posterior and lateral surface of the thyroid

## Intraoperative characteristics which identify the parathyroid glands

1. A normal parathyroid gland measures 4-6mm with a maximum diameter of no more than 8mm
2. It is surrounded by a capsule with fat around the capsule
3. The parathyroid has a characteristic yellowish brown or tan brown color
4. It is softer than a lymph node
5. The parathyroids become pale when devascularized and congested on venous injury
6. It has a smooth and regular surface (29)

## Techniques to identify and preserve the parathyroid glands

### Capsular dissection

Historically, the approach to thyroid surgery was to identify the recurrent laryngeal nerve early all along its length and remove tissue medial to the gland while attempting to preserve parathyroid blood supply, The issues with this approach were that the parathyroid glands were at risk of devascularization and the recurrent laryngeal nerve was also at risk due to the extensive dissection (2, 31). ‘Capsular dissection’ technique (**Figure 1**) in thyroid surgery is considered safe to preserve the recurrent laryngeal nerve and parathyroids and involves commencing the lateral dissection high on the thyroid gland dividing only the tertiary branches of the inferior thyroid artery. This safe ligation of the vascular supply close to the thyroid lobe is a way of preserving the blood supply of the parathyroid glands.

## Routine vs. selective identification of parathyroid glands

Conventional thyroid and central neck surgery mandates the routine identification of every parathyroid gland during the operation. Several studies have suggested that the risk of hypocalcaemia increases when less than 2 parathyroid glands are identified during surgery. However, routine identification poses several risks including- inadvertent damage to the vascularity during dissection, non-identification of the gland due to its absence in the orthotopic position, and increased surgical time spent in finding the glands (32). Functionality of auto-transplanted glands of dubious *in situ* viability has been questionable (33–35). Some recent studies have, however, showed that identification of greater number of parathyroid glands during surgery increased the risk of post-operative hypocalcaemia. This led several surgeons to adopt a selective approach of only looking for parathyroid glands in orthoptic positions along with a capsular dissection technique which should further protect the parathyroid glands from damage (36, 37). Selective identification preserves vascularity and also saves surgical time (1). However, the quality of studies, low overall incidence of permanent hypocalcaemia, and conflicting evidence available in the literature make it very difficult to draw any concrete recommendation about the choice of approach. A detailed anatomical knowledge and subjective assessment will help in the intraoperative preservation of parathyroids. Endocrine surgeons have been known to be particularly trained to understand the viability of the glands in situ (28). Presence of supernumerary glands should also be kept in mind while looking for parathyroids, the characteristics of which have been described above (33). Symptomatic permanent hypocalcaemia is uncommon if at least one viable parathyroid is retained (38). The use of magnification loupes of two and a half times can be of useful to identify the recurrent laryngeal nerve, the parathyroids, and vascular anatomy, however it does not replace meticulous dissection (39). An important technique is the careful dissection, skeletonization of the stem of the inferior thyroid artery, hence preserving parathyroid vasculature. Intraoperative testing includes immersing a part of the parathyroid in saline to see whether it sinks/floats or having frozen section analysis (40). The tubercle of Zuckerkandl acts as a pointer with the superior parathyroids superior to the tubercle and inferior parathyroids inferior to it (41). ‘Pinch, burn, and cut’ is another technique on the thyroid capsule to safeguard the nerve and the parathyroid (42–44).

## Inferior parathyroid gland preservation in central compartment lymph node dissection (CCLND)

**Figure 5:**
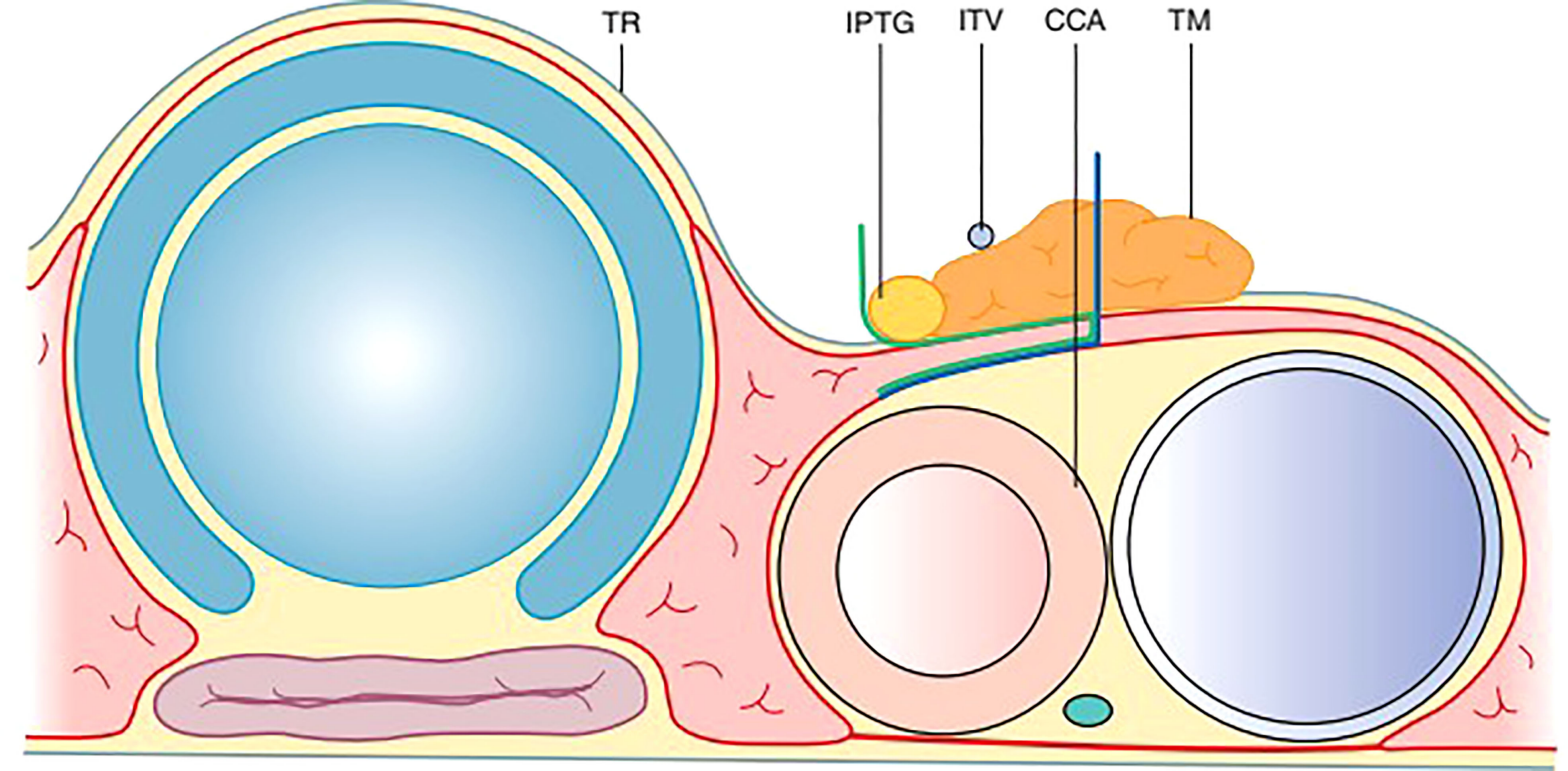
The TBP concept: Anatomy around the inferior pole of the thyroid lobe – Thymus, Blood vessel (inferior thyroid vein) and Parathyroid.

A layer of TBP (Thymus, blood vessels and parathyroids) (Xie): Thymus, blood vessels, and parathyroids are all arranged in one layer (**Figure 5**). This layer is connected to the layer covering the common carotid, innominate artery, and paratracheal nodes (28).

Embryologically, the thymus as well as the inferior parthyroid glands comes from the 3rd pharyngeal pouch. Both of them migrate toward the superior mediastinum as the fetus grows. The thymic sheath is connected to the thyroid by the thyrothymic ligament which forms the basis of the layer of TBP.

**Figure 6:**
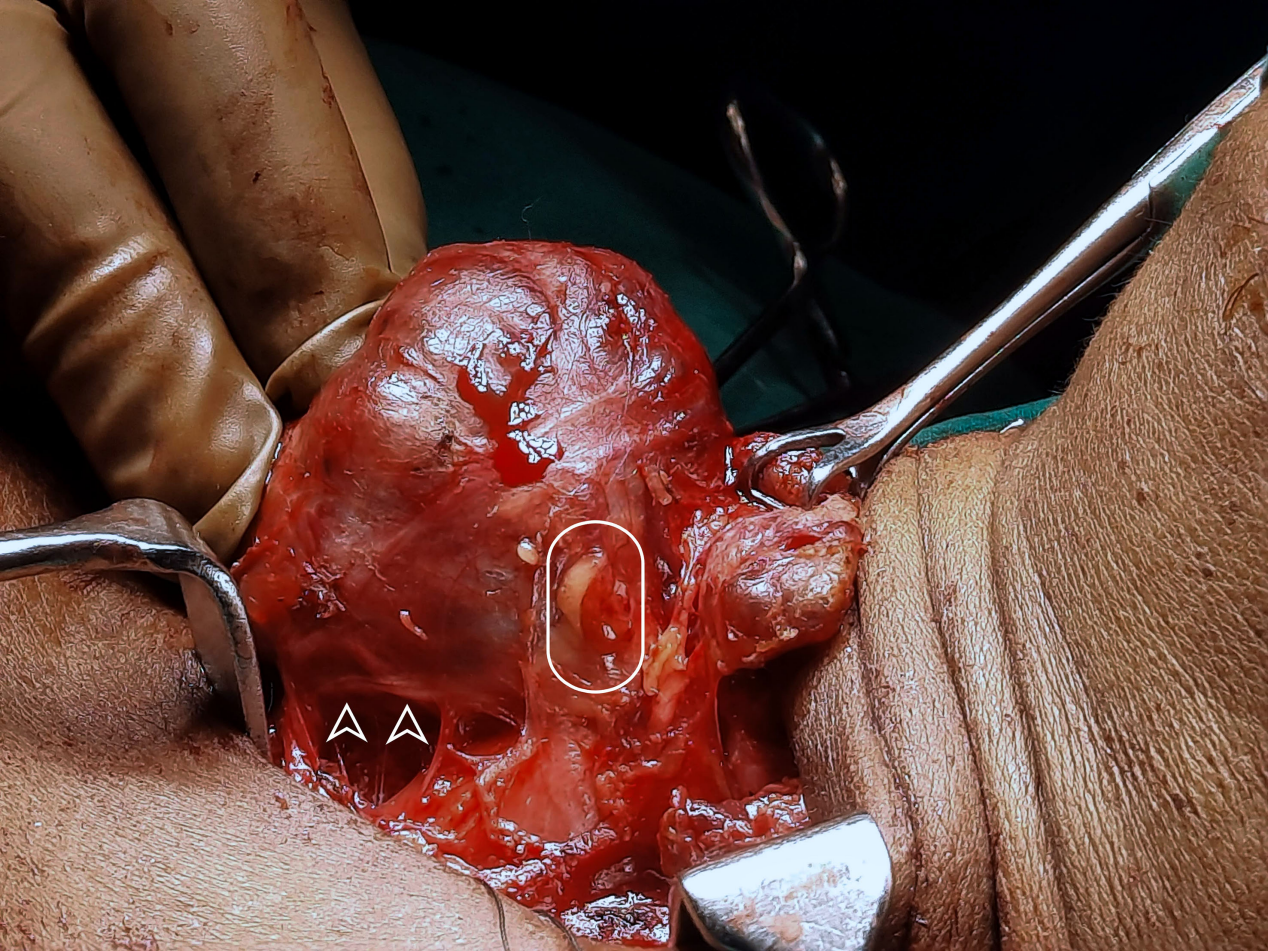
Intra-operative picture of thyroid lobe, the intact inferior parathyroid gland (oval) and the recurrent laryngeal nerve (arrowheads).

During central compartment neck dissection, the layer of TBP forms the lateral margin of the dissection. The area from Berry’s ligament to the thymus or brachiocephalic vessels forms the medial margin. The inferior thyroid artery is superficial to the TBP layer anterior to the common carotid artery. Hence, the layer of TBP, common carotid, and the recurrent laryngeal nerve are retracted laterally during the dissection of paratracheal nodes above and below the inferior thyroid artery. This concept of dissection led to significant improvement in the preservation of parathyroid *in situ* from 36 to 76% (**Figure 6**) (45, 46). Meticulous capsular dissection is another way to ensure parathyroid safety in situ.

## Energy dissection devices in relation to parathyroid preservation

The Scalpel is an important instrument in surgery used in dissection with the use of conventional methods of hemostasis. Hemostasis is generally achieved with a clamp after dissection at the superior pole. The dissection can cause traction injury to the vessels and damage the parathyroid. It can also shear the vessels leading to a clouded field. Newer devices like harmonic scalpels have minimal lateral thermal injury. They have better control over bleeding including hemostasis of vessels up to 5mm. The field of vision is clear for further dissection. This has reported reduced transient hypocalcaemia from 52 to 45%. Reduction in post-operative drainage in the drainage tubes, avoiding insertion of drainage tubes altogether and transient hypocalcaemia have been the major advantages of the use of harmonic scalpel in most studies (42, 47).

Another device used in recent days is the harmonic focus which functions similar to the harmonic scalpel with more focused energy. This has been compared in several studies with conventional techniques and found to have better results. A meta-analysis published in 2015 reported shorter operative time and hospital stay, lesser blood loss, and reduced hypocalcaemia and nerve injury (48). Despite the usage of these specialized instruments, certain procedures naturally have a higher rate of complications. Total thyroidectomy and central compartment neck dissections are associated with a higher complication rate as compared to hemithyroidectomy (49). The use of magnification loupes as mentioned earlier improves dissection. This has also been reported to reduce transient hypocalcaemia though it comes with a learning curve (39).

## Routine vs. selective autotransplantation

Most surgeons prefer a selective approach to autotransplantation, choosing only to autotransplant parathyroid glands which macroscopically appear of dubious viability or were inadvertently removed along with the thyroid specimen. Simply identifying parathyroid glands does not confirm viability as macroscopic assessment is highly subjective and inconclusive. Discoloured glands don’t necessarily confer non-viability while a lack of discoloration does not reliably predict an intact blood supply (50, 51). Routine parathyroid autotransplantation was therefore suggested by Delbridge et al. (52) where at least one parathyroid gland was routinely autotransplanted to guard against potential late ischemia in otherwise normal appearing glands. This approach was associated with a higher incidence of temporary hypocalcemia but had the potential to reduce incidence of permanent hypocalcaemia. Selective autotransplantation, however, remains the most widely practiced technique.

Nevertheless, the subjective nature and variable ability of surgeons to assess viability of parathyroid glands *in situ* warrants the need for more objective means of assessment (53).

## Parathyroid identification and fluorescence and other imaging, PARAFLUO trial

The technique of auto-fluorescence was originally found to be very accurate (29). The detection and preservation of parathyroid using autofluorescence by infrared cameras was nearly 100% accurate as demonstrated by Falco and McWade et al. (54, 55) Autofluorescence is indirect evidence of viable parathyroids while injection of fluorophores followed by detection of fluorescence proves to be more accurate. The injection of indocyanine green dye (ICG) followed by the detection of fluorescence with an infrared camera was studied by Suh and Lavazza et al. in 2015 and 2016 respectively (29, 56, 57). Methylene blue was another contrast agent proposed by Dudley in 1971 (58). Optical coherence tomography, a non-invasive imaging technique for the architectural characterization of parathyroids was proposed by Rubenstein et al. (59, 60) Fluorescence on the other hand was a well-established technique with a few drawbacks like difficulty in differentiation between fat and lymph nodes with a higher false positive rate. They were qualitative methods of detection necessitation quantification of fluorescence. ICG was approved by FDA in 1956 due to its lesser toxicity profile. A very minimal quantity (0.2mg/kg) was sufficient to detect fluorescence with an infrared camera 30 seconds to 2 minutes after injection. This proved to be a real-time, safe, and effective method of parathyroid identification and preservation (56, 57). However, there are contrasting reports too, that post-resection assessment of parathyroids by ICG has been more effective than real-time assessment, thus deeming it not fully competent (40). ICG has a wavelength of about 800nm and can detect parathyroids pre- and post-resection. However, the disadvantage of confusion with other structures which have similar uptake of the dye remains unaddressed. It can also soil the field due to leakage from the vessels (60). It also poses a significate learning curve as opposed to autofluorescence (61, 62). A randomized trial (PARAFLUO) of 245 patients published in 2020 suggested a significant reduction in autotransplantation rates from 13% to 3%, inadvertent parathyroidectomy from 12 to 2.5%, and transient hypocalcaemia from 21.7 to 9.1% (62).

## Carbon nanoparticles in LN dissection to preserve parathyroid glands

Carbon nanoparticle (CNP) suspension is a negative technique utilized for the preservation of important structures in thyroid surgery. They have an approximate size of 150 nm. Hence they act as lymphatic tracer that penetrates lymph vessels with a diameter of 120-500nm and not blood vessels that have a diameter of 20-50nm. The thyroid and lymph nodes stain black while the parathyroids and the nerve remain stainless. Parathyroids do not have lymphatic vessels developmentally and the nerves lack lymphatics. This only becomes an indirect technique to identify parathyroids (29). 0.1ml of CNP suspension is injected into superior and inferior thyroid poles away from vital structures. Adjacent blood vessels are kept away from the injection site. After 5 mins, the staining is observed. The rates of lymph node identification and parathyroid preservation were higher with this technique (63). Hagiwara et al. reported the first series using this technique in thyroid surgery (64). The rate of inadvertent parathyroidectomy was reduced by 34% according to a meta-analysis in 2022 (65).

Electrical Impedance spectroscopy (EIS) is another technique that seems promising. It is easy to master and can have wider applications. Adequate software for neck surgeries, suitable techniques to differentiate parathyroid from a lymph node, and also methods of identifying the drop in temperature would be required. Devascularized glands can prove a challenge since these are not visualized in both thyroid and parathyroid surgeries (66).

Laser Speckled Contrast Imaging (LSCI) is an alternative technique to use lasers to detect an interference pattern called the speckled pattern. The movement of blood cells within the vessels causes changes in the speckled pattern based on the flow. This helps us quantify the vascularity in real-time and does not need fluorescence (67). Mannoh et al. published a study suggesting the quantification using LSCI had a sensitivity and specificity of 87% and 84% respectively (68, 69).

## Parathyroid auto-transplantation

The first reported parathyroid autotransplant (PTAT) in a human being was performed by William Halsted in 1909. PTAT during thyroid surgery was first reported in 1926, when it was performed during partial thyroidectomy by Lahey (4). The first PTAT after total parathyroidectomy was described in 1968 (70).Viability of the auto-transplanted parathyroid was first reported in 1975 (5, 71). With time, people started acquainting themselves with the techniques of parathyroid autotransplantation and cryopreservation. Gradually, these methods gained popularity as measures to lower rates of hypocalcemia and hypoparathyroidism following total thyroidectomy.

Parathyroid tissue has certain attributes that enable it to survive, thrive and function after autotransplantation.

## Imbibition

Immediately after PTAT, the autotransplanted tissue survives by means of imbibition. This refers to the process of passive diffusion of water, oxygen and nutrients into the cells of the graft from the surrounding tissue fluid. The metabolic wastes diffuse out into the surrounding fluid. Grafts can survive by imbibition for up to a week after transplant. The key factors which influence survival of the graft by imbibition are the degree of perfusion of the graft bed and the size of the bits of tissue that are implanted. There are many reports (72–75) of successful PTAT after transplantation of the tissue within the belly of skeletal muscle, a tissue which is well-perfused and well-oxygenated.

The bits of tissue that are grafted should be small enough to survive the immediate post-operative period, before neo-vascularization. Successful PTAT has been reported with various sizes of the grafts (76–78).

Transplantation of bits of tissue with a volume of 1mm^3^ (1x1x1mm) ensures optimal survival of the tissue. With time, new blood vessels start to develop within the graft by virtue of angiogenesis. Various factors influence this process.

## Angiogenesis

The ability of the parathyroid tissue to induce angiogenesis has been shown *in vitro* (79) as well as *in vivo* (80). Vascular endothelial growth factor (VEGF) has been shown to contribute to the development of the angiogenic phenotype *in vitro* (81). Angiogenic activity was observed during the first post-operative week in athymic mice which were subjected to parathyroid auto-transplantation (82). In addition, re-innervation along newly built blood vessels has been demonstrated in transplanted parathyroid tissue 1 week post-operatively (83).

In a trans-species study (76), parathyroid tissue that was harvested from patients was cut into 2x2x1 mm pieces, which were subsequently transplanted into nude mice. Angiogenesis induced by these grafts was detectable by light microscopy on the 5^th^ day after the procedure. Newly grown microvessels were seen to be originating from host venules. Human iPTH was detected in plasma samples of the mice. So, it follows that the donor microvessels served as pathways for sprouting microvessels. Apparently, vascular ingrowths develop in about 10-20 days following implantation (84). Graft function mirrors this process as well and is reflected in serum PTH levels taken during this period.

## Site for transplantation

A well-perfused, vascular bed, like skeletal muscle belly, is necessary for autotransplantation in order for the graft to survive by imbibition before new blood vessels are formed by angiogenesis. Generally, the preferred sites for transplantation are the musculature of the forearm and the sternocleidomastoid muscle (72–75).

The advantage of employing the sternocleidomastoid is that another incision can be avoided. Successful PTAT into the brachioradialis has also been reported (78, 79, 85). While it requires a separate incision, it is much easier to assess functioning of the graft. PTAT into pectoralis major has also been reported (75). Funahashi, et al. employed this method in neck dissections. During neck dissection, when the sternocleidomastoid muscle is taped and freed completely, the blood supply to the muscle may be jeopardized, at least transiently. This may adversely affect graft viability. There is a report of successful pre-sternal subcutaneous autotransplantation of parathyroid glands (86).

So, while there are advocates of various sites within the body for implantation of harvested parathyroids, they all concur on the fact that belly of the skeletal muscle is a suitable site for PTAT.

## Method of transplantation

The smaller the graft, the more the chances of survival by imbibition. Billings and Milroy (85) have reported successful transplants in which the harvested parathyroid tissue was minced in iced Waymouth’s tissue culture medium. The resulting suspension was transplanted into the deltoid and brachioradialis muscles, by means of placement into pockets as well as by injection into the muscles.

In another study (78), the gland which was to be auto-transplanted was sliced into 30 pieces, each of size 1x1x3 mm. These bits were implanted into 30 pockets in the brachioradialis muscle of the forearm.

Testini, et al. (25) employed a technique in which the harvested parathyroid glands were sliced into 1x2mm pieces. One of them was sent for histological confirmation, while the rest of them were immediately autotransplanted into an intramuscular pocket in the sternocleidomastoid on the side of harvest. The site of transplantation was closed with a non-absorbable suture to prevent extrusion of the graft. The grafts were reported to be functional and viable.

Wells SA Jr., et al. (87), in their report about long-term follow-up after PTAT during thyroidectomy, reported that the harvested parathyroid gland was sliced into pieces of size 1x3mm, which were implanted into muscle pockets in the sternocleidomastoid muscle. These were found to be functioning in the long term.

Delbridge, et al. (88) reported successful PTAT after injecting a suspension of finely minced parathyroid tissue into the muscle bulk (Milroy technique). In this study, patients undergoing PTAT were divided into two groups. In the control group (implantation group), PTAT was performed in the conventional method of implanting bits of parathyroid tissue into muscle. In the test group (injection group), PTAT was performed by a novel method of injecting a suspension of finely minced parathyroid tissue into the muscle bulk (Milroy technique). Post-operative assessment included clinical assessment together with estimation of S. Calcium and intact parathormone (iPTH) immediately before the procedure and again on post-op days 1, 14 and 90. In both the groups, the procedure was followed by a fall in mean PTH levels, but it was significant in the implantation group. By 2 weeks, the calcium and iPTH levels had returned to baseline levels. None of the patients developed permanent hypoparathyroidism.

## Conclusion

Parathyroid function preservation remains one of the holy grails of thyroid surgery. This helps in limiting not just short term morbidity but also long-term impact on patient health, quality of life and health care costs. Evolution in surgical technique with capsular dissection, parathyroid identification and autotransplantation in routine use while adjuncts like surgical loupes and energy devices for fine dissection and haemostasis have helped improve outcomes. Questions remain on best practice in terms of selective versus routine parathyroid identification and autotransplantation. Technological advances utilising autofluorescence, laser speckled contrast imaging, electrical impedance spectroscopy and carbon nanoparticle uptake have provided promising potential direct and indirect methods of objective parathyroid identification (89). Nevertheless, parathyroid preservation during thyroid surgery continues to be an art where meticulous surgical technique, experience and expertise remain the mainstays in ensuring good outcomes.

## Disclosure

Patients who underwent thyroidectomy were informed about the possibility of usage of operative images for academic purposes and they have given their informed consent for the same.

## Author contributions

HR and SSR have jointly written the article. APR, TA and ZM have critically reviewed and edited the article. The latter trio have also provided valuable suggestions and revised the manuscript to its current format. All authors contributed to the article and approved the submitted version.
